# Tailoring luminescent and magnetic characteristics of Cu-doped CdSSe quantum dots for optomagnetic applications

**DOI:** 10.1039/d6ra04551k

**Published:** 2026-07-17

**Authors:** N. T. T. Hoan, N. X. Ca, L. A. Tuyen, N. Q. Hung, V. T. Nguyen, V. T. K. Lien, D. T. Hue, N. T. H. Nga, P. V. Duong, P. M. Tan

**Affiliations:** a Faculty of Fundamental and Applied Sciences, Thai Nguyen University of Technology Thai Nguyen Vietnam tanpm@ptit.edu.vn; b Institute of Sciences and Technology, TNU-University of Sciences Thai Nguyen Vietnam canx@tnus.edu.vn; c Center for Nuclear Technologies, Vietnam Atomic Energy Institute Ho Chi Minh City 70000 Vietnam; d Institute of Fundamental and Applied Sciences, Duy Tan University Ho Chi Minh City 70000 Vietnam; e Faculty of Natural Sciences, Duy Tan University Danang City 50000 Vietnam; f Institute of Theoretical and Applied Research, Duy Tan University Hanoi City 100000 Vietnam; g School of Engineering and Technology, Duy Tan University Da Nang 550000 Vietnam; h Faculty of Physics, Thai Nguyen University of Education Vietnam; i Faculty of Mechanical Engineering, Thuyloi University Hanoi city 100000 Vietnam; j Institute of Physics, Vietnam Acedemy of Sciences and Technology Vietnam; k Faculty of Fundamental Sciences, Posts and Telecommunications Institute of Technology Hanoi city 100000 Vietnam

## Abstract

Copper-doped ternary alloyed semiconductor quantum dots (QDs) are promising multifunctional nanomaterials owing to the synergistic combination of composition-dependent band-gap engineering and dopant-induced optical and magnetic functionalities. Despite extensive studies on Cu-doped binary CdS and CdSe QDs, investigations of Cu-doped ternary CdSSe QDs remain unexplored. In this work, CdSSe and Cu-doped CdSSe QDs with Cu concentrations ranging from 1 to 10% were successfully synthesized *via* a wet-chemical route. X-ray diffraction and Rietveld refinement analyses confirmed the formation of a single-phase zinc blende structure without detectable secondary phases. The diffraction peaks systematically shifted toward higher diffraction angles with increasing Cu content, accompanied by a reduction in the lattice parameter from 5.96 to 5.85 Å, indicating lattice contraction caused by the substitution of Cd^2+^ ions by smaller Cu^2+^ ions. Transmission electron microscopy revealed nearly spherical nanoparticles with average sizes increasing from approximately 3.5 to 5.0 nm upon Cu incorporation. The absorption peak exhibits a progressive blue shift, while the optical band gap increases from 2.48 to 2.83 eV as the Cu concentration increases from 0 to 10%. Cu doping creates defect-related radiation centers, leading to strong emission in the long wavelength region. The average lifetime increased sharply from 16.26 ns for undoped CdSSe QDs to 882.57 ns for CdSSe:Cu 10% QDs, demonstrating efficient carrier localization at Cu-related impurity levels. Magnetic measurements revealed that Cu doping significantly altered the magnetism of CdSSe QDs by generating local magnetic moments associated with the 3d state of Cu. This study demonstrates that Cu doping can simultaneously control the luminescence and magnetic properties of CdSSe QDs, making them attractive candidates for advanced optoelectronic and spintronic applications.

## Abstract 2

Schematic energy level diagram of CdSSe:Cu QDs.

## Graphical abstract

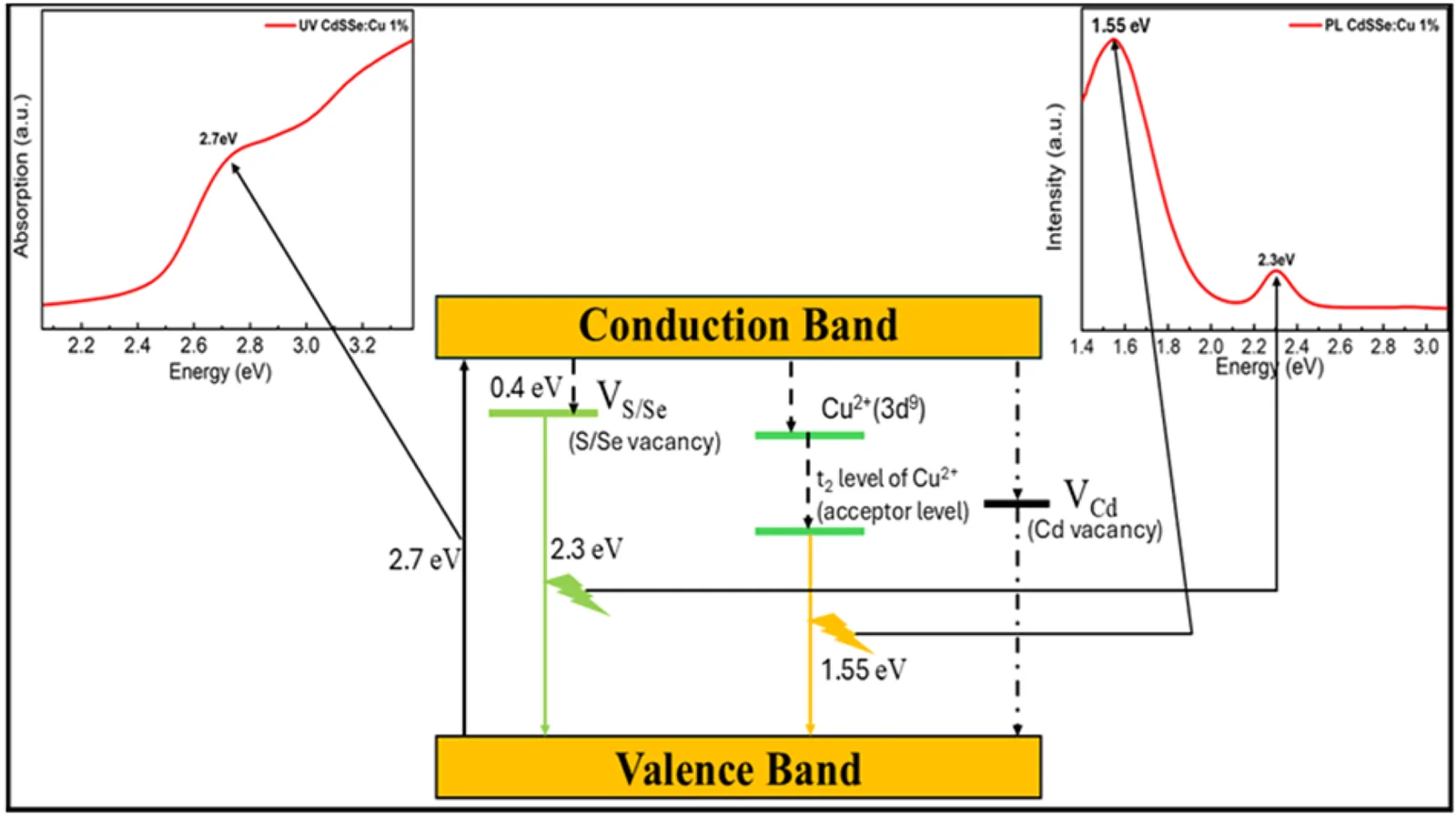

## 1. Introduction

In recent years, the rapid advancement of optoelectronic devices and spintronics technology has motivated scientists to research and develop semiconductor nanomaterials capable of flexibly tuning electronic states and electron spin ^1,2^. Devices such as high-efficiency solar cells, light-emitting diodes (LEDs), and biosensors require material band gap energy (*E*_g_) that can be precisely tuned.^3^ II–VI group semiconductor QDs such as CdS and CdSe are widely applied in optoelectronics due to their strong absorption and emission capabilities in the visible region. In particular, they exhibit a strong quantum confinement effect, which allows their optical and electronic properties to be tuned by changing their particle size or composition.^4^ When the crystallite size decreases below the exciton Bohr radius, the electronic structure of the material changes from continuous bands to discrete energy levels, allowing for the tuning of the band gap energy and optical properties through changes in particle size and shape. However, binary CdS and CdSe QDs have a limited emission range because their emission wavelength tunability is primarily achieved through particle size control.^5^ To overcome this limitation, researchers have focused on studying ternary alloyed CdS_*x*_Se_1−*x*_ QDs.^6,7^ By varying the S/Se ratio, the emission spectra of CdSSe QDs can be tuned from the blue region to the red region without changing the particle size.^7^

To tune and enhance the optical and magnetic properties of alloyed ternary QDs, many studies have focused on transition metal doping. The incorporation of transition metal ions into the host crystal lattice can create shallow or deep trap energy levels within the forbidden band gap, which serve as new radiative recombination centers. In previous studies, various metal ions such as Co, Ni, Cu, and Mn have been doped into semiconductor QDs. Ni doping into CdTeSe QDs not only increases the luminescence time up to 580 ns but also creates magnetic properties at room temperature in the material.^8^ Similarly, Gd-doping in CdTeSe QDs enhances the photoluminescence lifetime by more than 10-fold compared to undoped CdTeSe QDs, as the dopants create deep trap centers that reduce the spatial overlap between the electron and hole wavefunctions.^8,9^ Meanwhile, Co doping into CdS helps enhance the radiative recombination process at low concentrations (approximately 2%).^1^

Among the transition metals investigated, copper (Cu) has attracted significant interest and is being extensively researched due to its outstanding optical properties. Cu ions can exist in either Cu^+^ or Cu^2+^ oxidation states within the semiconductor crystal lattice. Furthermore, Cu incorporation has the capability to enhance the photoluminescence quantum yield (QY) of the host material. The study by S. Wageh and coworkers on the Cu-doped system reported an exceptionally high QY, reaching up to 63%, which was attributed to the emergence of a permanent optically active hole at the Cu mid-gap energy level.^10^ Structurally, since the ionic radius of Cu^2+^ (0.73 Å) is significantly smaller than that of Cd^2+^ (0.97 Å), the Cu doping process typically induces lattice compression, and increases the micro-strain, which consequently affects the energy band structure. Oluwafemi *et al.*, in their study of l-cysteine-capped Cu-doped CdSe QDs, observed a significant redshift of the emission peak and an increase in particle size with increasing Cu concentration.^11^ For the CdS system, studies have demonstrated that Cu doping reduces the excitonic emission intensity and enhances trap-state emission originating from surface defects and Cu-related centers.^12^ In addition to its influence on optical properties, Cu doping can also modify the magnetic property of semiconductor QDs. The sequential and selective generation of Cu vacancies on the surface of multicomponent QDs is essential for overcoming the kinetic energy barriers.^13,14^ The incorporation of Cu ions into the host lattice introduces localized magnetic moments associated with their partially filled 3d orbitals. These magnetic moments enhance the exchange interactions between charge carriers and magnetic ions. Depending on the Cu concentration, oxidation state, and distribution within the crystal lattice, Cu-doped semiconductor QDs may exhibit either paramagnetic behavior or weak ferromagnetism at room temperature. Furthermore, lattice distortion and defect states induced by Cu incorporation can facilitate carrier-mediated magnetic coupling, leading to improved magnetic ordering.

Although Cu-doped CdS and CdSe QDs have been widely investigated, to the best of our knowledge, no study has yet reported the synthesis and systematic characterization of Cu-doped ternary CdSSe QDs. Compared to binary CdS and CdSe QDs, ternary CdSSe alloy QDs possess a significant advantage because their electronic structure can be tuned by particle size and alloy composition. Varying the Se/S ratio allows for adjustment of the energy gap across a wide spectral range while maintaining relatively large particle sizes and good crystallinity. When combined with Cu doping, the CdSSe alloy provides two independent tuning parameters: composition and impurity concentration. This allows for simultaneous tuning of the energy band structure, defect-related emission, and magnetic properties. Such synergistic control is considerably more difficult to achieve in binary Cu-doped CdS or CdSe QDs. The combination of the ability to tune the energy gap based on the composition of CdSSe alloy systems and the optical and magnetic functionalities generated by Cu doping is expected to provide new opportunities for tuning the physical properties of semiconductor nanomaterials. Therefore, the systematic synthesis and study of Cu-doped CdSSe QDs is a novel and important research topic. Cu-doped CdSSe QDs opens up significant prospects for the development of multifunctional nanomaterials, which can be optimized for specific applications ranging from displays to photovoltaics.

## 2. Experimental section

### 2.1. Materials

The materials used in the preparation of CdSSe QDs and Cu-doped CdSSe QDs included: CdO powder (99.5%), Se powder (99, 98%), S powder (99, 98%), Copper(ii) chloride (CuCl_2_), 1-octadecene (ODE, 90%), trioctylphosphine (TOP, 97%), axit oleic (OA, 90%), *n*-hexane và isopropanol (98%). All reagents were purchased from Sigma-Aldrich and employed without additional purification.

### 2.2. Synthesis

#### 2.2.1. Synthesis of CdSSe alloyed QDs

The CdSSe QDs were synthesized in a three-necked flask under an inert Argon (Ar) gas flow. The anion precursors were prepared by dissolving Sulfur (S) powder and Selenium (Se) powder in a mixed solvent of ODE and TOP at 100 °C under continuous stirring. The cationic precursor was synthesized in a three-neck flask by stirring CdO with OA and ODE at a temperature of 200 °C for 60 minutes. Subsequently, the temperature was elevated. When the temperature in the three-neck flask reaches 250 °C, the solution containing S^2−^ and Se^2−^ precursors is swiftly injected into the solution containing Cd^2+^ ions, and the reaction is maintained for 60 minutes. The obtained solution containing CdSSe QDs was mixed with isopropanol and centrifuged at 14.000 rpm for 5 minutes to remove remnants. Subsequently, the sample was redispersed in *n*-hexane for further measurements.

#### 2.2.2. Synthesis of Cu doped CdSSe alloyed QDs

**Fig. 1:**
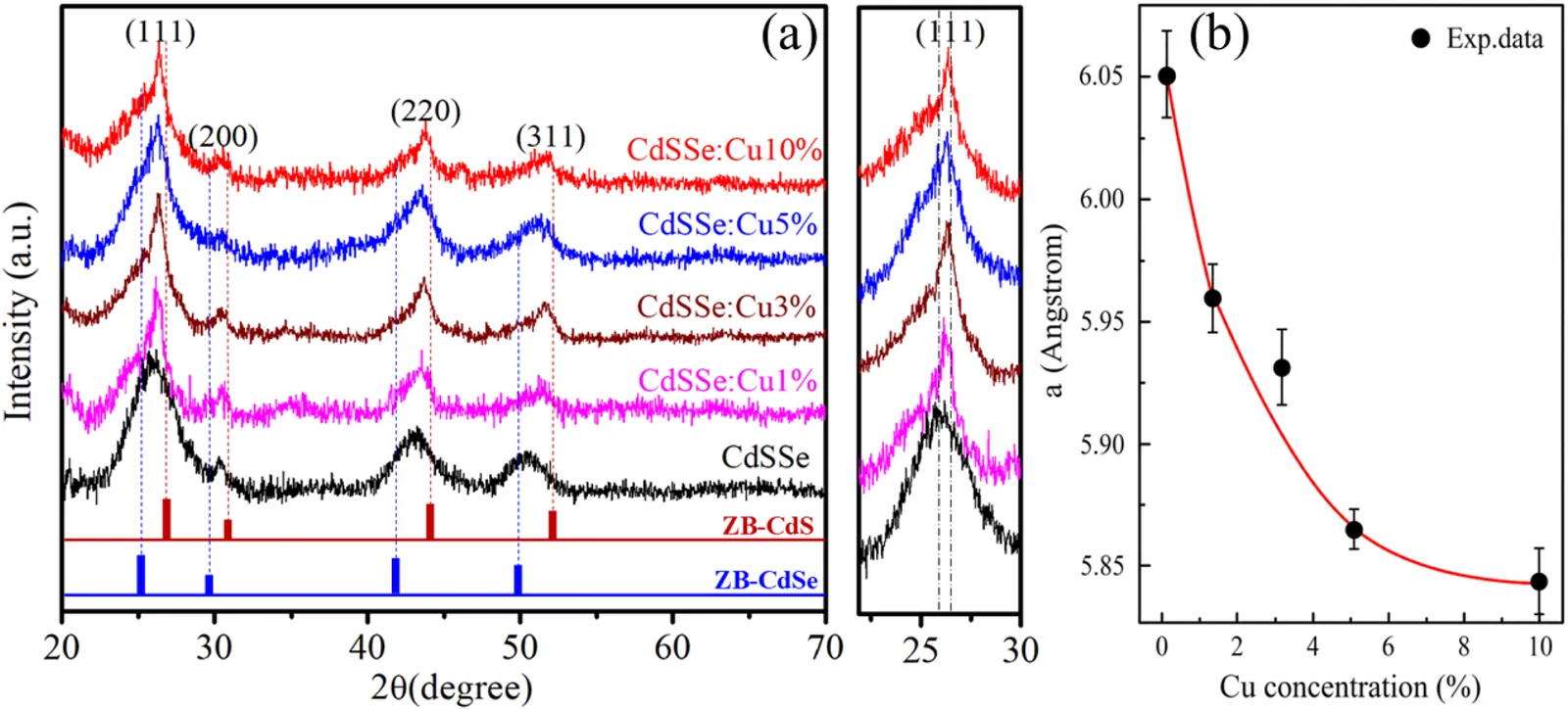
(a) X-ray diffraction pattern of undoped CdSSe and 1–10% Cu-doped CdSSe QDs, (b) the dependence of the lattice parameter on Cu concentration.

Cu-doped CdSSe QDs were synthesized in a manner similar to the undoped CdSSe QDs. CuCl_2_ salt was stirred in OA and ODE at 100 °C for 30 minutes to obtain a solution containing Cu^2+^ ions. The amount of CuCl_2_ salt was taken such that the Cu/Cd molar ratios were 0.01, 0.03, 0.05, and 0.1. Once the solution containing CdO, OA, and ODE reached 200 °C for 60 minutes, the Cu^2+^-containing solution was injected into the three-neck flask (Fig. 1). Subsequently, when the temperature reached 250 °C, the solution containing S^2−^ and Se^2−^ precursors was rapidly injected into the three-neck flask, triggering instantaneous nucleation. The reaction was maintained for 60 minutes. The sample purification process was performed similarly to that of the CdSSe QDs.

### 2.3. Measurements

The crystalline phases of the prepared samples were characterized by X-ray diffraction (XRD) using a Bruker D8-Advance diffractometer with Cu-Kα radiation (*λ* = 1.5406 Å). Transmission electron microscopy (TEM) observations were carried out on a JEOL JEM-1400 microscope operated at an accelerating voltage of 120 kV to evaluate particle morphology and size. The surface morphology and microstructural characteristics were further investigated by field-emission scanning electron microscopy (FESEM) using a Regulus 8100 system. The elemental composition and spatial distribution were analyzed *via* energy-dispersive X-ray spectroscopy (EDX) with an XFlash 6130 detector attached to the FESEM instrument. Optical absorption properties were examined using a Jasco V-770 UV-vis spectrophotometer. Photoluminescence (PL) emission spectra and fluorescence decay profiles were recorded on an FLS1000 spectrometer. The emission spectra were collected over the wavelength range of 390–900 nm with a step interval of 0.5 nm, while the excitation wavelength was maintained at 375 nm.

## 3. Results and discussion

### 3.1. Structure and morphology study

Fig. 1a presents the XRD patterns of CdSSe and CdSSe:Cu QDs with Cu concentrations of 1, 3, 5, and 10%. For the undoped CdSSe QDs, four characteristic diffraction peaks are observed at 2*θ* ∼ 25.86°, 30.12°, 43.13°, and 50.68°, which can be indexed to the (111), (200), (220), and (311) planes of the zinc blende (ZB) cubic structure, confirming the formation of a crystalline CdSSe phase. It can be observed that the diffraction peaks of the CdSSe QDs lie between the diffraction peaks of CdS and CdSe, indicating that the elements Cd, S, and Se are evenly distributed in the CdSSe QDs.^15,16^ The Cu-doped CdSSe QDs retain the same ZB crystal structure, as evidenced by the presence of the (111), (200), (220), and (311) reflections. The weak intensity of the (200) diffraction peak can be attributed to its relatively low structure factor compared with the preferentially oriented (111) plane.

Furthermore, the nanoscale crystallite size induces significant peak broadening, while the intense and broadened (111) reflection partially overlaps with the (200) diffraction signal, resulting in a substantial reduction in the observed intensity of the (200) peak.^17–19^ The broad diffraction peak of the (111) plane in the undoped CdSSe QDs, with a full width at half maximum (FWHM) of approximately 3.37°, indicates the presence of small crystallite sizes. When Cu enters the CdSSe crystal lattice, the (111) peak becomes narrower, suggesting an increase in crystallite size. As the Cu concentration increases up to 10%, the XRD diffraction peaks gradually shift by approximately 0.5° toward higher 2*θ* angles. According to Bragg's law, this shift indicates a decrease in the interplanar spacing. This behavior can be attributed to the incorporation of smaller Cu ions into the host lattice, which induces local lattice distortion and compressive strain. The peak shift also confirms that Cu is introduced into the CdSSe crystal structure rather than forming a separate secondary phase.

The Debye–Scherrer formula was used to calculate the average crystallite size of the QDs:1
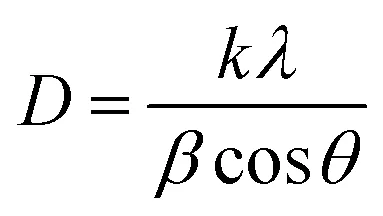
where: *D* is the crystallite size, *k* = 0.9 is a constant, *λ* is the X-ray wavelength, *θ* is the Bragg angle, and *β* is the width at half maximum (FWHM) of the diffraction peak. The results are shown in Table 1.

**Table 1:** The lattice parameters and crystallite sizes of the samples were obtained from Rietveld refinement

Sample	2*θ* (°)	*a* (Å)	*β* (°)	*D* (nm)
CdSSe	25.86	5.96	3.37	2.42
CdSSe:Cu 1%	26.17	5.89	2.96	2.76
CdSSe:Cu 3%	26.28	5.87	3.03	2.69
CdSSe:Cu 5%	26.32	5.86	3.05	2.68
CdSSe:Cu 10%	26.37	5.85	3.01	2.71

Fig. 1b shows that the lattice constant *a* of the CdSSe:Cu QDs decreases with increasing Cu dopant concentration. This trend may be attributed to the partial substitution of Cd^2+^ ions (0.97 Å) by smaller Cu^2+^ ions (0.73 Å), resulting in lattice contraction of the CdSSe QDs, in agreement with Vegard's law. As a result, a slight shift of the diffraction peaks toward higher 2*θ* angles is observed (Table 1).

**Fig. 2:**
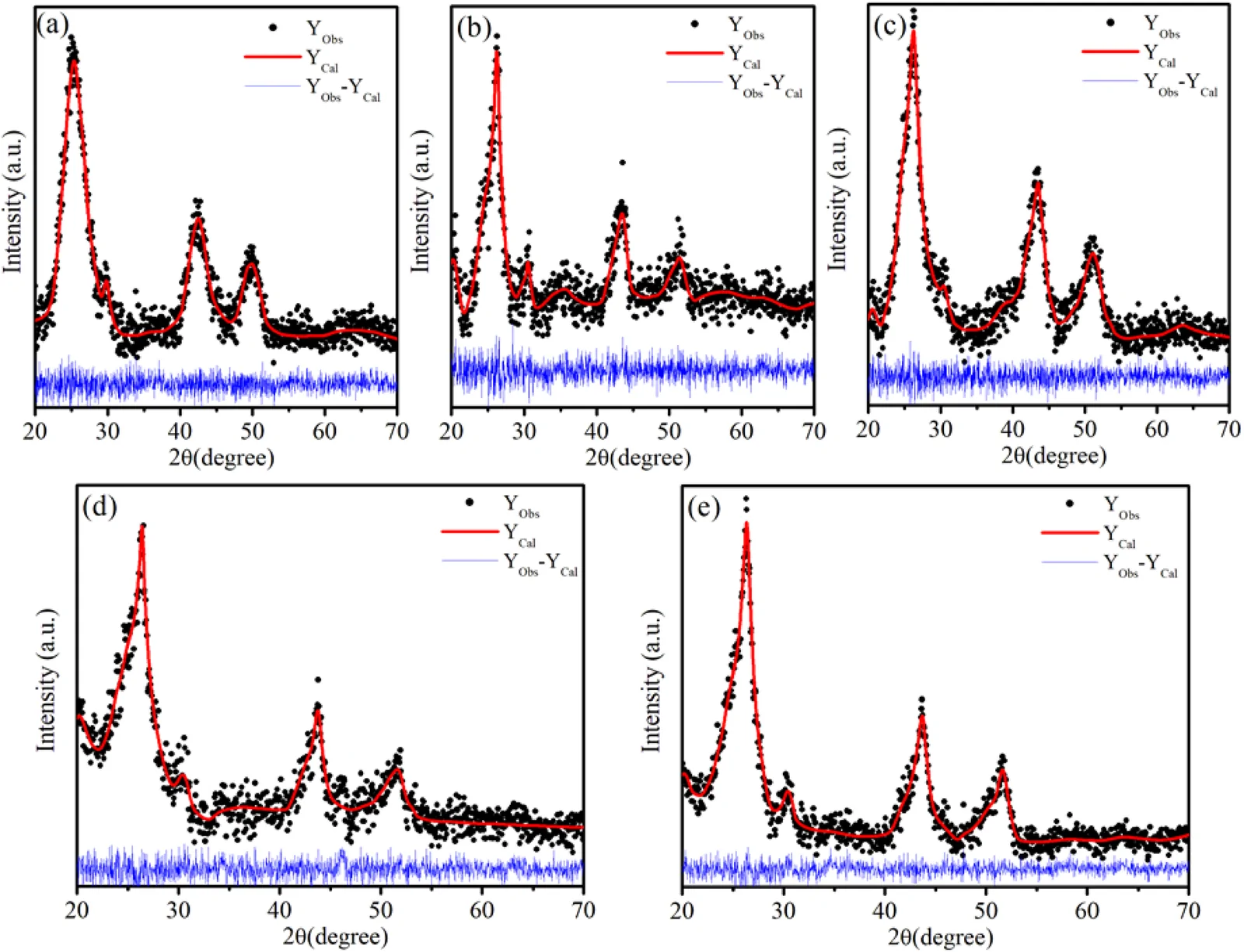
Rietveld refinement of XRD patterns for Cu-doped CdSSe QDs at doping concentrations: (a) 0, (b) 1, (c) 3, (d) 5, and (e) 10%.

The Rietveld refinement patterns of pure and Cu-doped CdSSe QDs with different Cu concentrations are shown in Fig. 2a–e. For the structural analysis of the XRD patterns, the Rietveld refinement was carried out using the FullProf analytical software.^20^ The diffraction peaks of all samples were refined using the Pseudo–Voigt profile function.^21^

The refinement results reveal that all samples retain the same crystal structure, with no evidence of secondary impurity phases. The good agreement between the (*Y*_obs_) and (*Y*_cal_) patterns, while the residual curves (*Y*_obs_ − *Y*_cal_) remain relatively small, confirms the reliability of the refinement process. The refined lattice parameters are summarized in Table 1. The obtained refinement results are in good agreement with previously reported studies.^6,22^

**Fig. 3:**
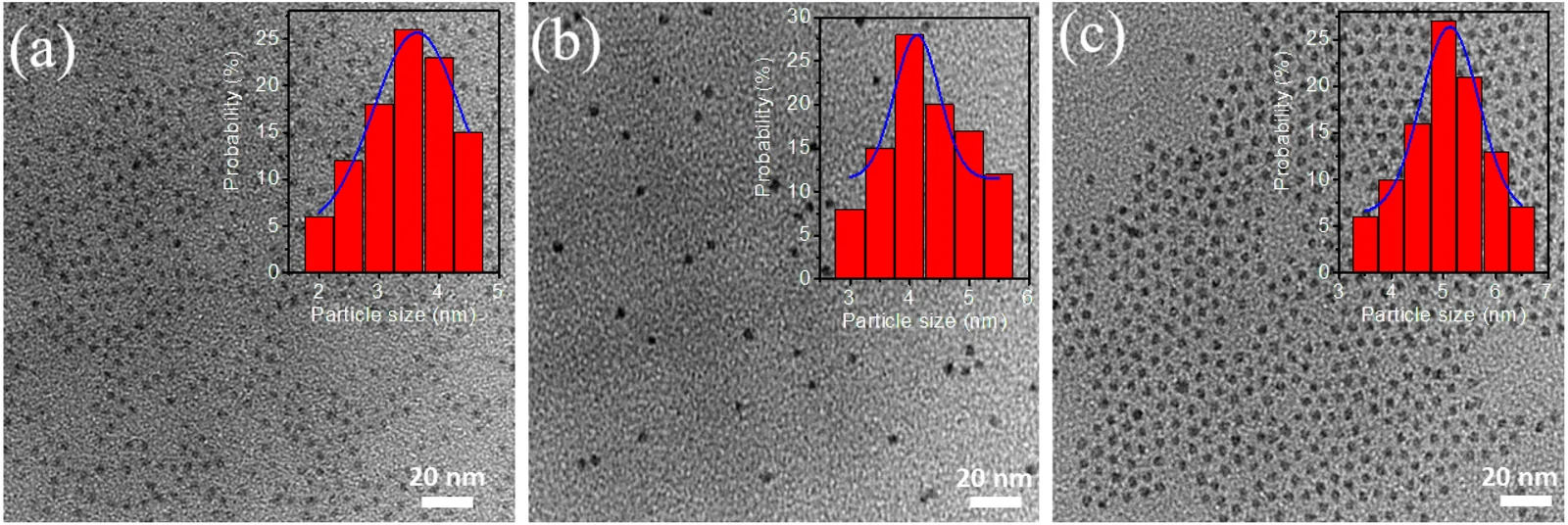
TEM images of (a) CdSSe, (b) CdSSe:Cu 5% and (c) CdSSe:Cu 10% QDs.

TEM images of CdSSe, CdSSe:Cu 5%, and CdSSe:Cu 10% QDs are shown in Fig. 3. The images reveal that the QDs are nearly spherical in shape and exhibit a relatively uniform size distribution. The average particle sizes of CdSSe, CdSSe:Cu 5%, and CdSSe:Cu 5% QDs were 3.5, 4.0, and 5.0 nm, respectively. This indicates that the particle size gradually increases with increasing Cu concentration.

### 3.2. Compositional and vibrational spectroscopic analysis

**Fig. 4:**
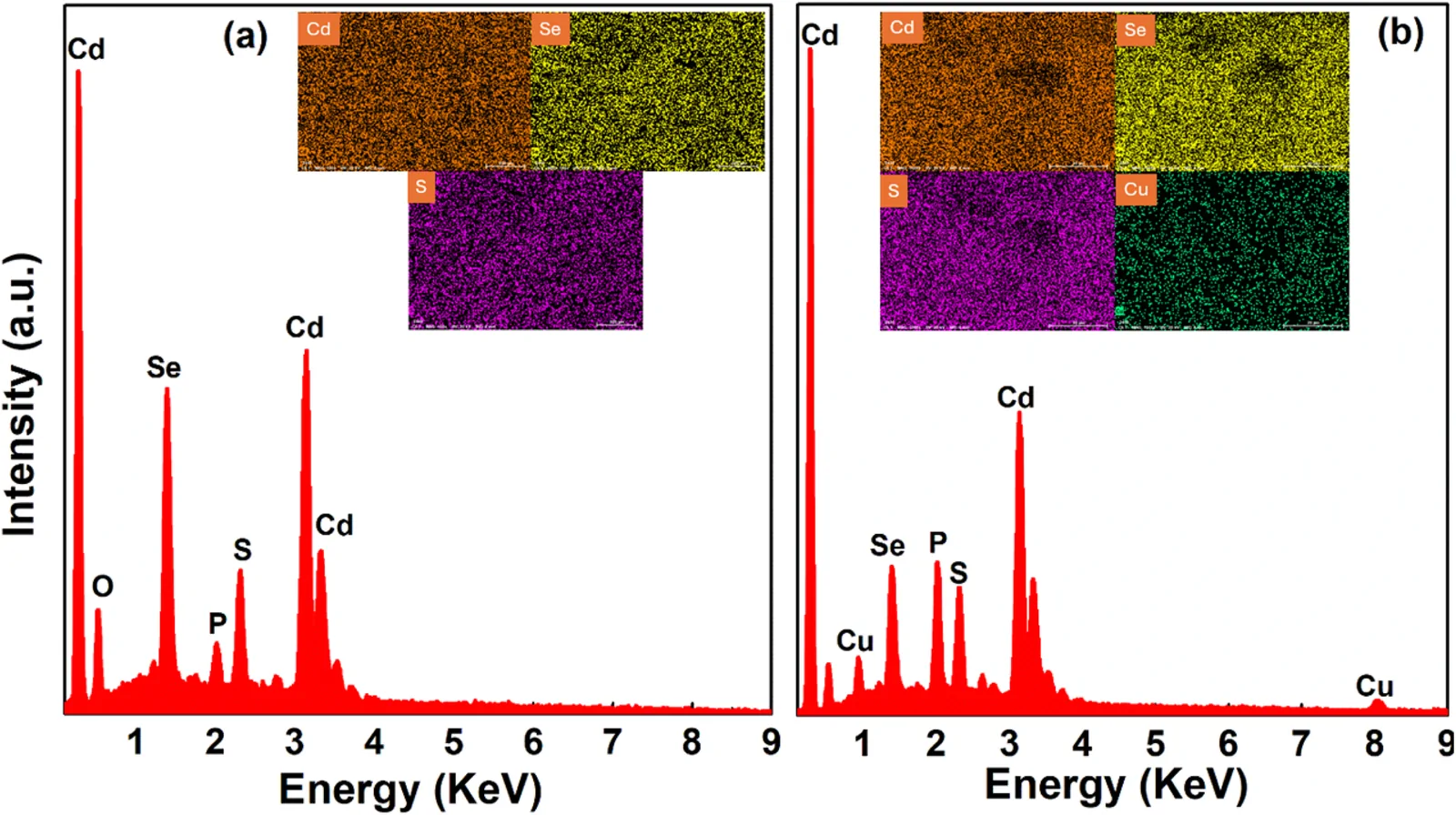
EDX spectra and EDX elemental mapping of (a) undoped CdSSe, and (b) CdSSe:Cu 10% QDs.

After centrifugation and washing, the undoped and Cu-doped CdSSe QDs were dispersed in *n*-hexane. The samples were drop-cast onto glass slides and dried naturally to investigate the elemental composition of the QDs by EDX measurement. Fig. 4 presents the EDX spectra and EDX elemental mapping of CdSSe and CdSSe:Cu10% QDs. The EDX spectrum of CdSSe QDs exhibits characteristic peaks corresponding to Cd, S, and Se. The spectrum of CdSSe:Cu 10% QDs displays additional peaks at 0.93 keV and 8.04 keV, which are attributed to Cu. The percentage composition by weight and element is summarized in Table 2. The atomic percentage of Cd decreases progressively with increasing Cu content, from 60.3% in CdSSe QDs to 49.3% in CdSSe:Cu 10%. This trend suggests that Cu^2+^ ions partially substitute Cd^2+^ ions within the CdSSe host lattice, in agreement with the structural analysis. The actual Cu concentration determined from the EDX spectra is lower than the nominal value (Table 2), suggesting that some Cu^2+^ ions did not substitute for the Cd^2+^ ions, and the excess Cu ions were likely removed during the washing process. Additionally, minor signals of O and P are detected in the EDX spectra, which can be attributed to residual organic species, such as trioctylphosphine (TOP) and oleic acid (OA), remaining after the purification process. The elemental mapping of CdSSe and CdSSe:Cu 10% QDs is shown in the inset of Fig. 4. The mapping results reveal that Cd, S, Se, and Cu are homogeneously distributed throughout the sample. Notably, Cu is detected only in the CdSSe:Cu 10% sample and is absent in the undoped CdSSe.

**Table 2:** Atomic and mass percentages of elements in undoped CdSSe and CdSSe:Cu QDs derived from EDX spectra

Sample	Weight (%)				Atomic (%)			
	Cd	S	Se	Cu	Cd	S	Se	Cu
CdSSe	74.26	5.88	19.86	—	60.3	16.74	22.96	—
CdSSe:Cu 1%	68.83	6.9	23.72	0.55	53.87	18.94	26.42	0.77
CdSSe:Cu 3%	68.34	6.3	23.97	1.38	53.81	17.4	26.87	1.92
CdSSe:Cu 5%	66.17	7.3	23.75	2.78	50.7	19.61	25.91	3.78
CdSSe:Cu 10%	64.69	6.59	21.36	7.37	49.3	17.59	23.18	9.93

**Fig. 5:**
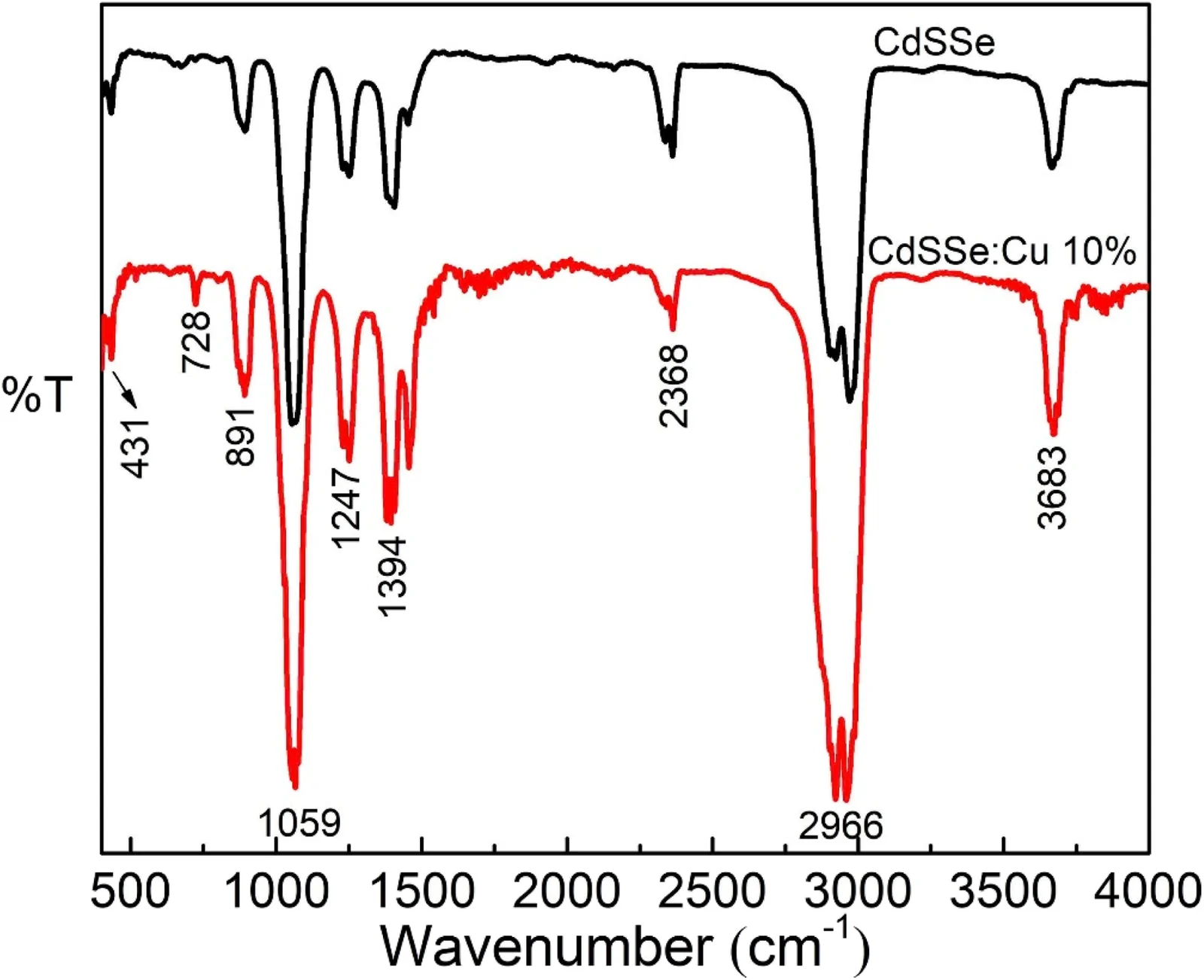
FTIR spectra of undoped CdSSe and CdSSe:Cu 10% QDs.

The functional groups of the materials were investigated using FTIR spectroscopy. The FTIR spectra of CdSSe and CdSSe:Cu 10% QDs, recorded in the range of 400–4000 cm^−1^ with a resolution of 0.96 cm^−1^, are shown in Fig. 5. The absorption peak at 3683 cm^−1^ is attributed to O–H stretching vibrations, indicating the presence of a small amount of adsorbed moisture on the sample surface. The band at 2966 cm^−1^ corresponds to C–H stretching vibrations,^23^ which can be associated with residual organic species from the synthesis process. The appearance of these vibration peaks can be attributed to OA and TOP molecules capping the QDs surface. These ligands help passivate surface states, stabilize the nanocrystals, regulate crystal growth, and suppress particle agglomeration. In the low-wavenumber region, several studies have reported that absorption bands around ∼700 cm^−1^ are characteristic of Cd–S bonding,^24,25^ while vibrations in the range of 500–740 cm^−1^ have also been assigned to Cu–S stretching modes.^26^ The appearance of a band at 728 cm^−1^ in the Cu-doped samples may be associated with the formation of Cu–S bonds. This observation shows that Cu^2+^ ions are integrated into the CdSSe crystal lattice, forming a doped alloy system.

### 3.3. Optical studies

**Fig. 6:**
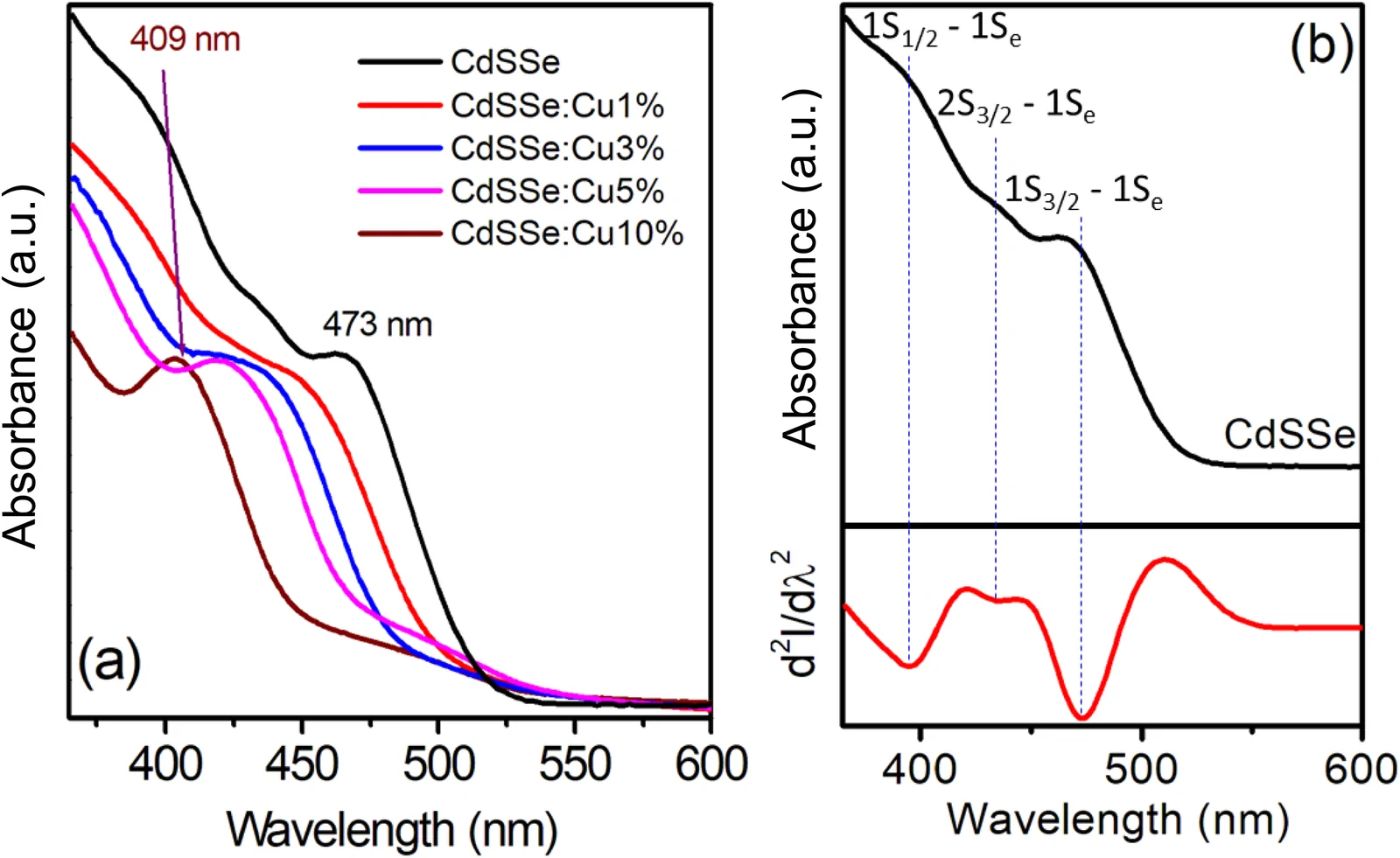
(a) UV absorption spectra of CdSSe, 1%, 3%, 5% and 10% Cu-doped CdSSe QDs. (b) UV absorption spectrum of CdSSe QDs and its quadratic derivative.

The optical properties of semiconductor QDs depend on size-induced quantum confinement effects, as well as defect or impurity energy levels located within the forbidden band gap. Fig. 6a illustrates the absorption spectra of the CdSSe and Cu-doped CdSSe QDs with concentrations of 1, 3, 5, and 10%, respectively, under an excitation wavelength of 375 nm. Fig. 6a shows that the QDs have distinct absorption peaks at 473 nm (for CdSSe), 460 nm (for CdSSe:Cu 1%), 445 nm (for CdSSe:Cu 3%), 427 nm (for CdSSe:Cu 5%), and 409 nm (for CdSSe:Cu 10%).

As the Cu concentration increases, the absorption peak gradually shifts toward shorter wavelengths, indicating a pronounced blue shift. In semiconductor QDs, such a blue shift is commonly attributed to a reduction in particle size and the resulting enhancement of the quantum confinement effect. However, TEM analysis (Fig. 3) reveals that the average particle size increases with increasing Cu content, suggesting that the observed blue shift cannot be explained by size-dependent effects. The shift is more likely associated with Cu-induced modifications of the electronic structure of the CdSSe host lattice.

To accurately identify the excitonic transitions, the second derivative of the absorption spectra was calculated, as presented in Fig. 6b. This method enhances weak spectral features, allowing for clear differentiation of excited states. The first three derivative minima correspond to the fundamental interband transitions involving the quantized valence- and conduction-band states, which are assigned to the 1S_3/2_–1S_e_, 2S_3/2_–1S_e_, and 1S_1/2_–1S_e_^27^ excitonic transitions, respectively. These transitions originate from the lowest confined hole states to the first electron state and are characteristic of the quantum-confined electronic structure of CdSSe QDs. The energy band gap of the samples was determined using Tauc's relation between the absorption coefficient and the incident photon energy:^28^

**Fig. 7:**
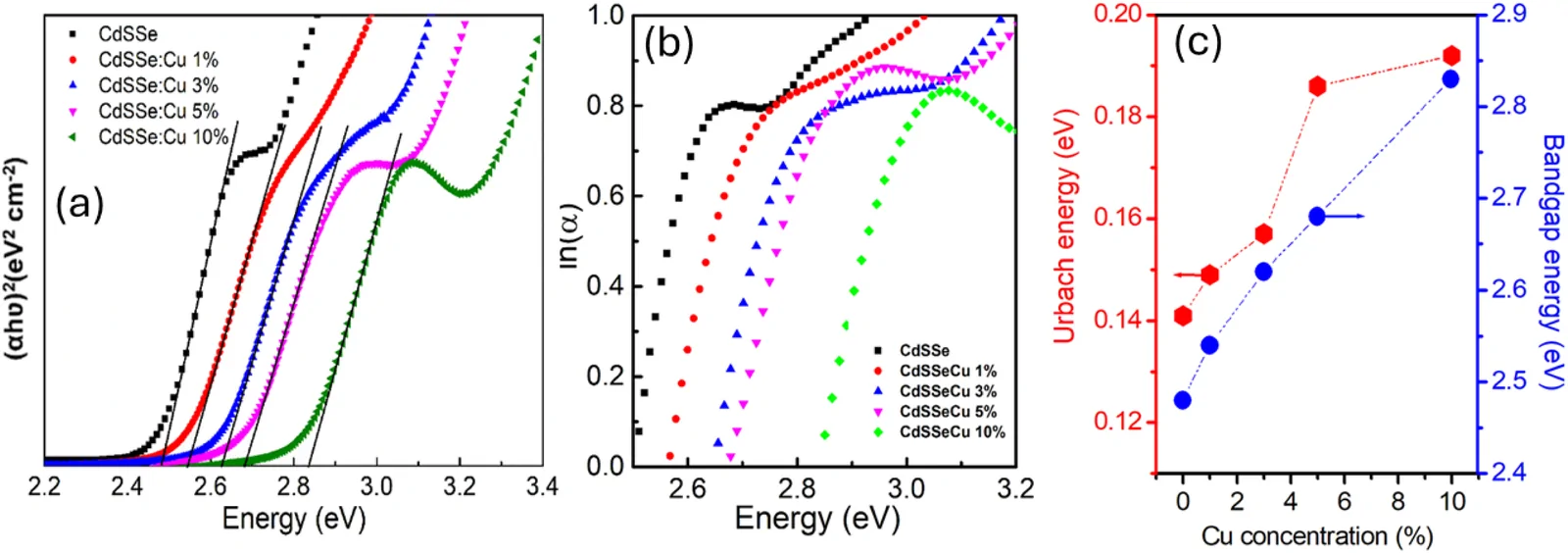
(a) (*αhν*)^2^*versus hν* plots for undoped CdSSe, 1%, 3%, 5% and 10% Cu-doped CdSSe QDs. (b) Urbach energy of undoped and Cu-doped CdSSe QDs with Cu concentrations ranging from 1% to 10%. (c) Dependence of the Urbach energy on Cu concentration in undoped and Cu-doped CdSSe QDs.

2 (*αhυ*) = *A*(*hν* − *E*_g_)^*n*^

where *A* is a constant, *E*_g_ is the band gap energy of the QDs, and *n* depends on the type of electronic transition. For the CdSSe QDs, *n* = 1/2 corresponds to the allowed direct transition. Fig. 7a illustrates the plots of (*αhν*)^2^*versus* the incident photon energy *hν* for the undoped and Cu-doped CdSSe QDs. Extrapolation of the straight portion of the curve to the energy axis gives the *E*_g_ values of the samples. The *E*_g_ values of the CdSSe, CdSSe:Cu 1%, CdSSe:Cu 3%, CdSSe:Cu 5%, and CdSSe:Cu 10% QDs are obtained as 2.48, 2.54, 2.62, 2.68, and 2.83 eV, respectively. This blue shift in *E*_g_ may be attributed to changes in the electronic structure induced by Cu doping, such as the introduction of localized states or modification of band edge positions. When Cu^2+^ ions partially substitute Cd^2+^ ions, impurity-related electronic states can be introduced into the crystal lattice, altering the positions of the conduction band and valence band. In addition, hybridization between Cu orbitals and the host electronic states may modify the band structure and effectively widen the band-gap.

Another possible contribution is the Burstein–Moss effect. Cu incorporation may increase the concentration of free carriers, causing the low-energy states near the bottom of the conduction band to become occupied. Optical transitions can occur only to higher-energy unoccupied states, leading to an apparent increase in the optical band gap and a corresponding blue shift of the absorption spectrum. Furthermore, Cu doping may induce local compositional and structural changes in the CdSSe lattice. The incorporation of Cu ions can affect the distribution of S and Se atoms and introduce lattice distortion, thereby modifying the local chemical environment. The combined effects of electronic structure modification, carrier concentration enhancement, and local compositional variation are likely responsible for the observed blue shift.

The absorption tail was observed to be raised as the Cu content increased (Fig. 7a). This phenomenon can be attributed to the introduction of Cu-related localized states and lattice imperfections, which increase the density of electronic states near the band edges. Therefore, optical transitions involving these localized states become more probable, leading to enhanced sub-band-gap absorption and a more pronounced Urbach tail. To further evaluate the influence of Cu doping on the degree of structural disorder, defects, and the formation of localized states near the band edge, the Urbach energy (*E*_u_) of the CdSSe:Cu QDs was determined from the absorption tail of the UV-vis spectra. The *E*_u_ is an important parameter for evaluating the degree of structural disorder and the distribution of localized states near the band edge in semiconductor QDs. The *E*_u_ of the CdSSe:Cu QDs was determined from the linear region of the plot of ln(*α*) *versus* photon energy (*hν*) (Fig. 7b) based on the Urbach rule:^29,30^3
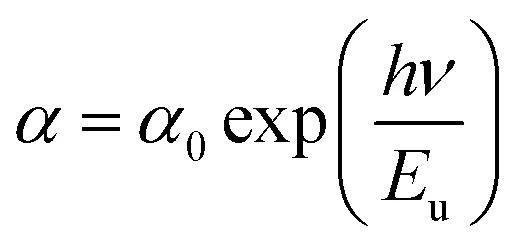


Taking the natural logarithm of both sides yields:4
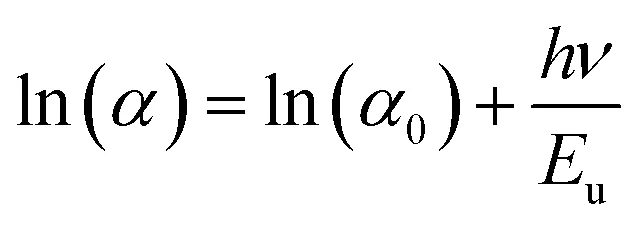
where *α* is the absorption coefficient, *α*_0_ is a constant, and *E*_u_ is the Urbach energy. The values *E*_u_ were calculated as the reciprocal of the slope of the linear region of the ln(*α*) *versus hν* plots; the results are presented in Fig. 7c. The results indicate that the *E*_u_ values increase with increasing Cu dopant concentration, rising from 0.141 eV (for undoped CdSSe) to 0.192 eV (for CdSSe:Cu 10% QDs). This increase indicates that the incorporation of ions into the host lattice has led to an increase in defect states and localized energy levels within the band gap. These states lead to the broadening of the absorption tail and an increase in the *E*_u_.

**Fig. 8:**
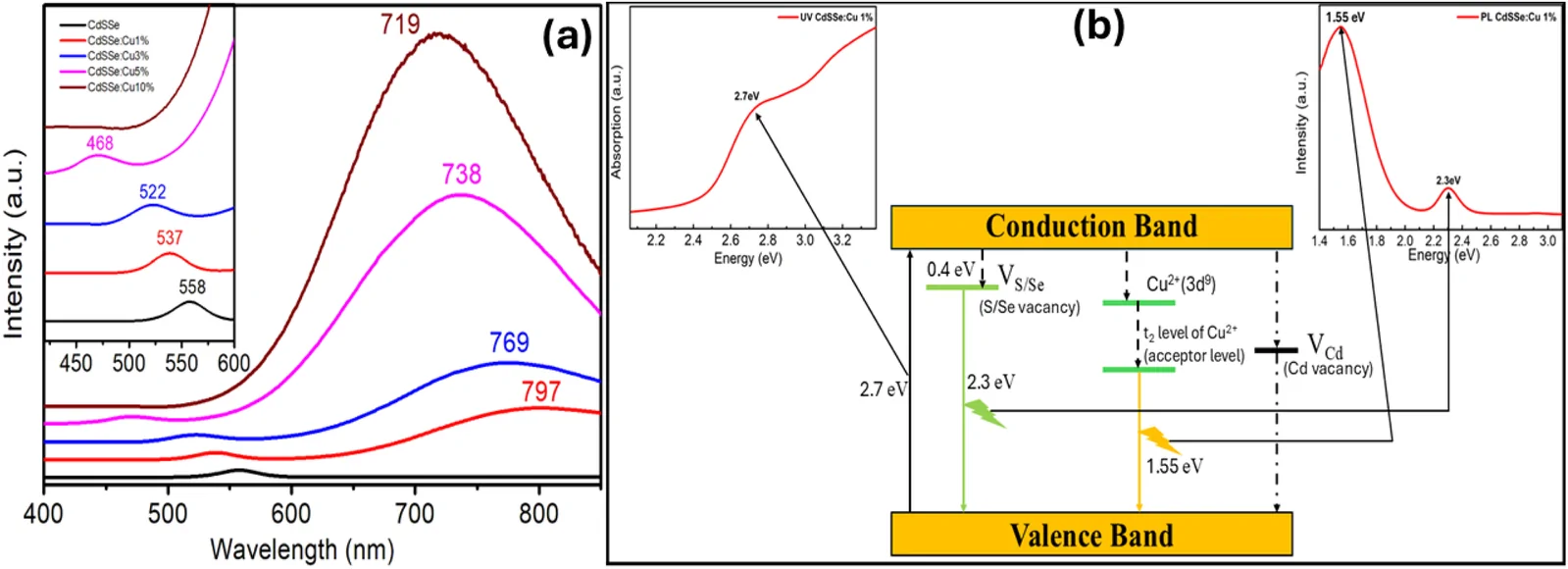
(a) PL spectra of undoped and Cu-doped CdSSe QDs with Cu concentrations ranging from 1% to 10%. (b) Schematic energy level diagram of CdSSe:Cu 1% QDs.

To investigate the effect of Cu doping on the luminescence behavior of CdSSe QDs, the PL spectra of undoped and Cu-doped samples were recorded using an excitation wavelength of 375 nm with excitation and emission slit widths of 2 nm (Fig. 8a). The PL spectrum of the undoped CdSSe QDs exhibits a distinct emission peak in the blue-green region (558 nm). This peak is attributed to the excitonic recombination process between the conduction band minimum (CBM) and the valence band maximum (VBM). The short-wavelength emission peak of the CdSSe QDs is narrow and well-defined, demonstrating that the material possesses a narrow size distribution and high crystallinity.^31^ Long-wavelength emission peaks were almost absent in the PL spectra of the CdSSe QDs. This indicates that during the synthesis of CdSSe QDs, the TOP and OA solvents effectively passivated the surface, thereby suppressing non-radiative recombination, energy trap levels, or crystal lattice defects.^7,10,32^

For the CdSSe:Cu QDs, the PL spectra exhibit two emission peaks, including a short-wavelength peak (*E*_high_) associated with excitonic emission and a long-wavelength peak (*E*_low_). The *E*_low_ emission peak is attributed to lattice defects, imperfections, and Cu-related trap states. The PL spectra of the CdSSe:Cu QDs have been intensity-normalized to the *E*_low_ peak. For Cu concentrations of 1%, 3%, and 5%, the *E*_high_/*E*_low_ values are located at 537/797 nm, 522/769 nm, and 468/738 nm, respectively. Fig. 8a shows that the intensity of *E*_low_ is much higher than that of *E*_high_, indicating that the energy levels created by Cu within the band gap have formed new recombination pathways and gradually become dominant over the band-to-band emission. This mechanism is illustrated in the energy band diagram of the CdSSe:Cu 1% QDs (Fig. 8b), in which the localized d-states of Cu^2+^ ions act as carrier trap centers and promote the low-energy radiative recombination process. A similar phenomenon has been reported in Cu-doped CdS QDs,^17^ where levels act as deep energy states within the forbidden gap, allowing conduction band electrons to recombine directly with holes at the Cu level without the participation of valence band holes. Notably, as the Cu concentration increases to 10%, the PL spectra of the CdSSe:Cu QDs only exhibit the *E*_low_ peak, while the excitonic peak disappears completely. This indicates that at high Cu dopant concentrations, CuS or Cu_2_S phases may form on the surface of the host material, quenching the emission of the host material.^33^ Previous studies on Cu-doped II–VI semiconductor QDs, such as ZnCdSe:Cu^33^ and CdTeSe:Cu,^34^ have shown that the *E*_high_ emission is suppressed at higher dopant concentrations, while impurity-related emission becomes dominant. Notably, both the *E*_high_ and *E*_low_ peaks of the CdSSe and CdSSe:Cu QDs exhibit a blue shift as the Cu concentration increases. The blue shift of both emission peaks of CdSSe as Cu concentration increases can also be explained by sp–d exchange interactions between local d electrons of Cu^2+^ ions and conduction and valence band electrons of the host lattice. As Cu^2+^ replaces Cd^2+^ in the lattice, this interaction shifts the conduction band edge to higher energies, leading to an increase in band gap energy.^35^ The blue shift of the PL spectra upon Cu doping into the host lattice has been previously reported by Ca *et al.*^33^ for Cu-doped ZnCdSe nanoparticles. Sivasankar *et al.* also noted the widening of the energy gap from 1.75 eV to 1.79 eV as the Cu doping concentration in the host CdSe lattice increases.^35^ The authors suggest that this blue shift is due to the increased carrier concentration resulting from the incorporation of Cu into the host material.

Fig. 8b illustrates the schematic energy-level diagram and the possible radiative transitions in CdSSe:Cu 1% QDs. The optical band gap of the CdSSe:Cu QDs was estimated to be approximately 2.7 eV from the UV-vis absorption spectrum, corresponding to the electronic transition from the valence band (VB) to the conduction band (CB). After excitation, electrons are promoted to the CB and subsequently captured by defect-related intermediate states associated with sulfur/selenium vacancies (*V*_S/Se_) and Cu^2+^ impurity levels. The Cu dopant introduces localized acceptor states, particularly the *t*_2_ level of Cu^2+^ (3d^9^), within the forbidden band gap of CdSSe. The broad emission band centered around 2.3 eV is attributed to the radiative recombination of electrons trapped at sulfur/selenium vacancy-related states with holes in the valence band. The lower-energy emission at 1.55 eV originates from the transition involving the Cu^2+^ acceptor level and the valence band. In addition, Cd vacancies (*V*_Cd_) may act as deep trapping centers, facilitating non-radiative relaxation processes and influencing the luminescence efficiency of the QDs. The proposed energy-level model provides a reasonable explanation for the observed absorption and photoluminescence behaviors of the Cu-doped CdSSe QDs.

### 3.4. Photoluminescence decay curves

**Fig. 9:**
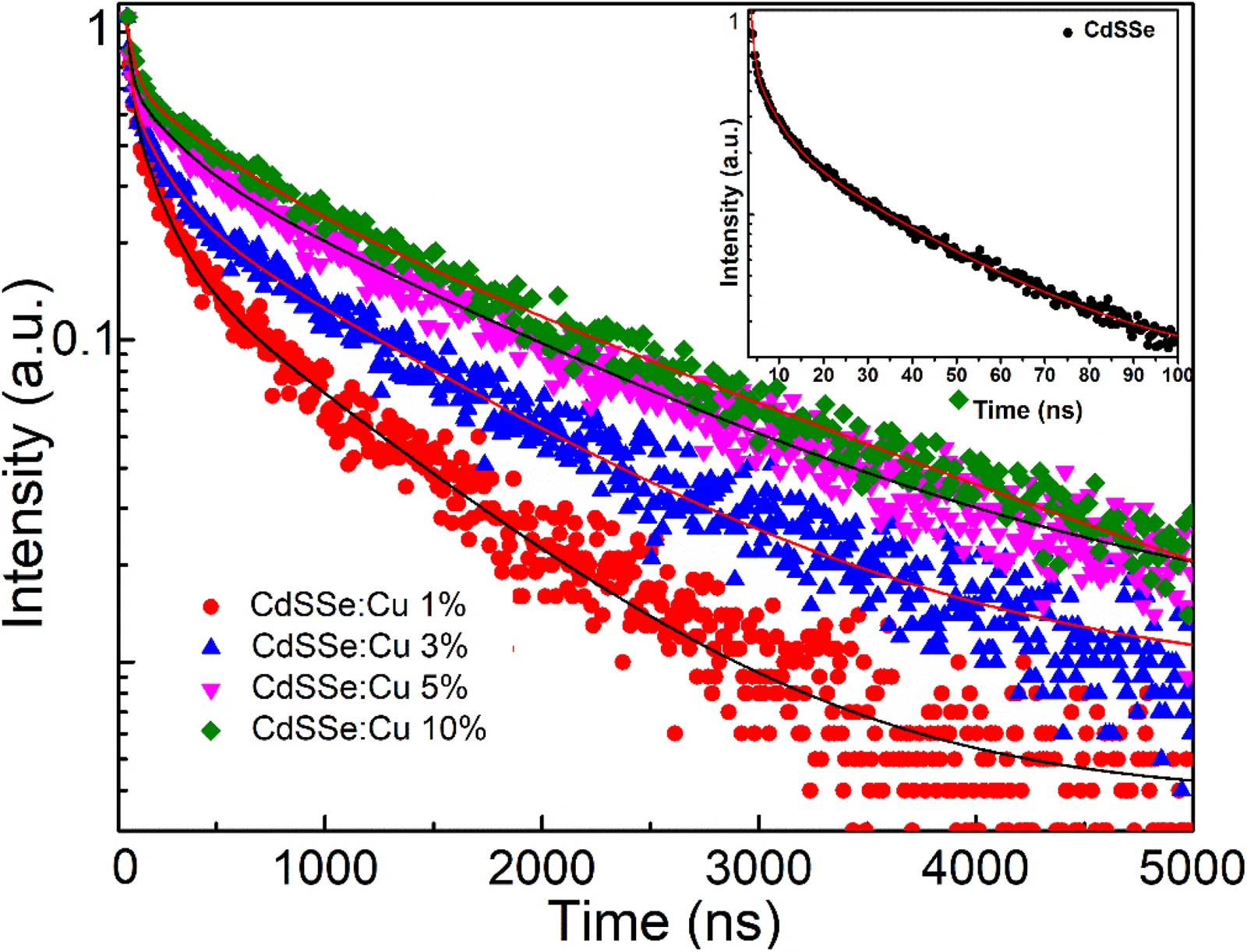
PL decay kinetics recorded at the emission peak energy of representative undoped CdSSe and CdSSe:Cu QDs.

Fig. 9 illustrates the time-resolved photoluminescence spectra of the CdSSe, CdSSe:Cu 1%, CdSSe:Cu 3%, CdSSe:Cu 5%, and CdSSe:Cu 10% QDs, recorded at their strongest emission peaks under an excitation wavelength of 375 nm. The CdSSe QDs (with an emission peak at 558 nm) exhibit a very rapid PL signal decay, being completely extinguished at approximately 100 ns. Meanwhile, the CdSSe:Cu QDs show a slower PL decay and are completely extinguished at about 5000 ns. The PL decay curves of the QDs obey multi-exponential functions, reflecting the complex nature of the luminescence process.^36,37^ The experimental values were fitted to the following equation:^37,38^5
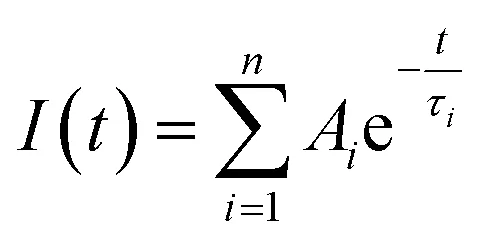
where *n* is the number of components contributing to the decay process. For this study, the CdSSe and CdSSe:Cu QDs are well-fitted with *n* = 3. *A*_*i*_ and *τ*_*i*_ represent the contribution percentage and the lifetime constant of each decay component, respectively. The average lifetime 〈*τ*〉 was calculated according to the following equation:^8,39^6
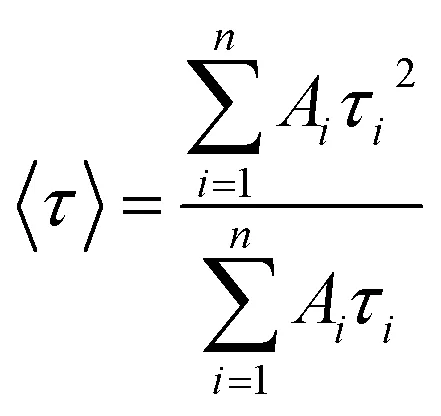


The lifetimes of the samples were calculated, and the results are presented in Table 3. The lifetime constants of the CdSSe QDs are *τ*_1_ = 0.62 ns, *τ*_2_ = 6.44 ns, *τ*_3_ = 42.1 ns, and the average lifetime is *τ* = 16.26 ns. Wherein, *τ*_1_ is ascribed to the band-edge excitonic emission. The *τ*_2_ component is attributed to emission from deep traps related to surface defects, while the *τ*_3_ component is assigned to the dark exciton states and/or Cu-related localized impurity states.^40,41^ When Cu is doped into the CdSSe host lattice, the lifetimes of the samples increase significantly compared to the undoped CdSSe QDs. Specifically, the PL lifetimes of the CdSSe:Cu QDs are 100.97 ns (for CdSSe:Cu 1% with emission at 797 nm), 190.1 ns (for CdSSe:Cu 3% with emission at 769 nm), 671.33 ns (for CdSSe:Cu 5% with emission at 738 nm), and 882.57 ns (for CdSSe:Cu 10% with emission at 719 nm), respectively. When Cu is doped into the CdSSe host lattice, Cu^2+^ ions create impurity energy levels within the forbidden band gap, typically acting as acceptor levels situated just above the valence band maximum. Following photoexcitation, electrons from the conduction band can recombine with holes trapped at the Cu impurity level instead of recombining directly with holes in the valence band. Due to the highly localized nature of the Cu impurity state, while the conduction band electrons are delocalized, the spatial wavefunction overlap between the electron and the hole localized at the Cu site is significantly reduced.^42–44^ This reduces the recombination probability, leading to much longer lifetimes compared to excitonic recombination processes. As a result, the PL lifetimes of the Cu-doped CdSSe QDs are significantly longer than those of pure CdSSe. The phenomenon of increased lifetime with higher Cu concentrations was also observed in ZnCdSe:Cu QDs^33^ and CdSe:Cu.^45^ The authors generally suggest that the increase in lifetime upon Cu doping is related to the localized Cu impurity states.

**Table 3:** Experimental values of time constants for CdSSe:Cu (0, 1, 3, 5, and 10% Cu) alloyed QDs

Sample	*τ*_1_ (ns)	*τ*_2_ (ns)	*τ*_3_ (ns)	〈*τ*〉(ns)
CdSSe	0.62	6.44	42.1	16.26
CdSSe:Cu 1%	16.82	127.33	808.74	100.97
CdSSe:Cu 3%	15.52	148.35	1042.19	190.1
CdSSe:Cu 5%	20.56	215.72	1275.42	671.33
CdSSe:Cu 10%	37.54	413.75	1603.36	882.57

### 3.5. Magnetic studies

**Fig. 10:**
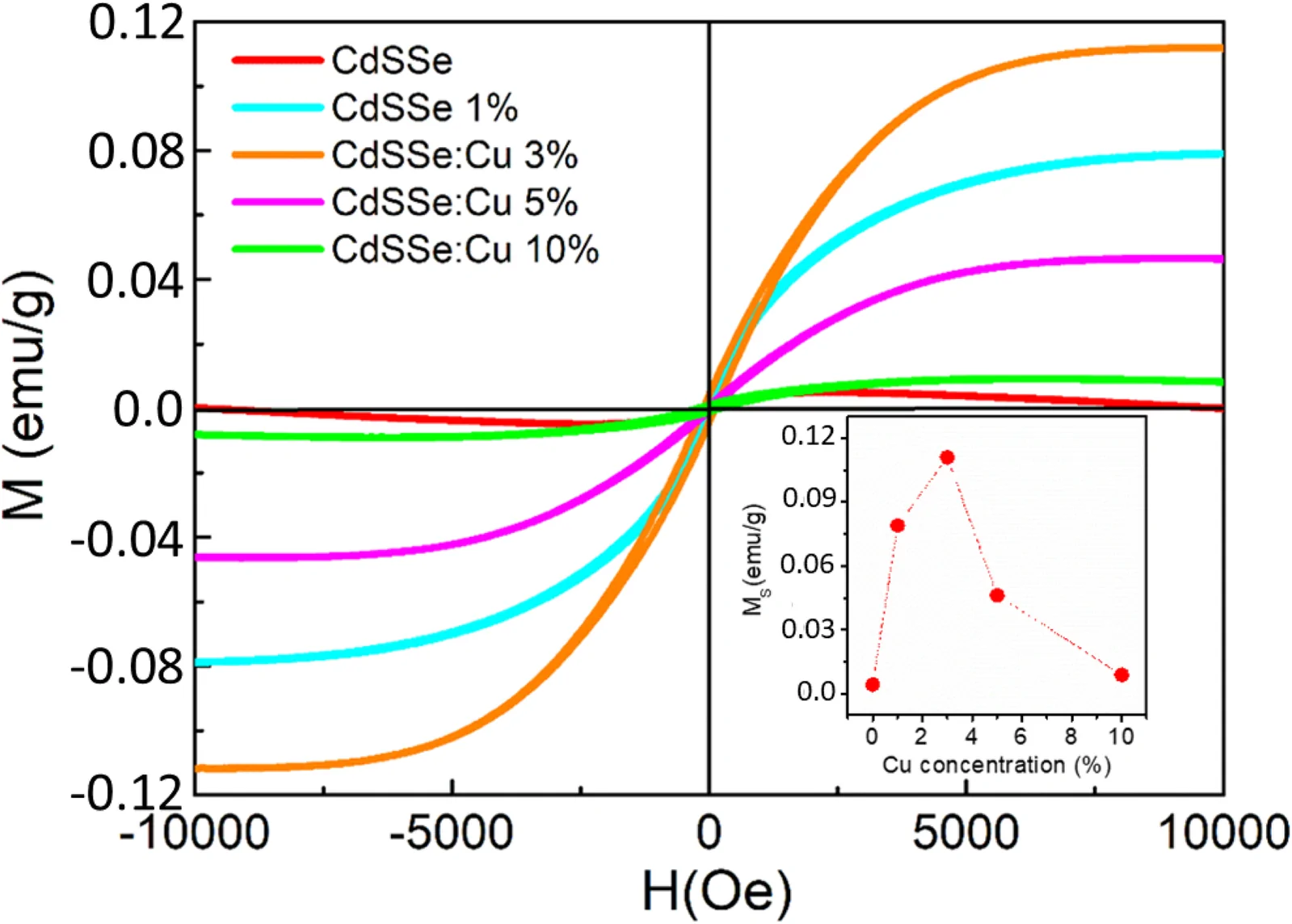
Room temperature M-H plots of CdSSe:Cu (with 0, 1, 3, 5, 10% Cu) QDs.

After centrifugation and washing, the undoped and Cu-doped CdSSe QDs were dispersed in *n*-hexane and dried naturally. The magnetic properties of the dried samples were investigated at room temperature. The magnetic properties of the CdSSe and Cu-doped CdSSe QDs were investigated at room temperature using field-dependent magnetization measurements (M–H curves). Fig. 10 illustrates the magnetic hysteresis (M–H) loops of undoped CdSSe and Cu-doped CdSSe QDs with Cu concentrations varying from 0–10%. The undoped CdSSe QDs exhibit an extremely weak magnetic response over the entire applied magnetic-field range. The very narrow M–H curve indicates the absence of magnetic hysteresis, suggesting that pure CdSSe QDs have negligible intrinsic magnetism and essentially behave as a non-magnetic semiconductor at room temperature. CdSSe QDs are generally regarded as intrinsically diamagnetic semiconductors.^8,9,34^ However, the undoped CdSSe QDs in this study exhibit a weak but detectable magnetic response, suggesting that the observed magnetism does not originate from long-range magnetic ordering of the host lattice. It is likely associated with intrinsic point defects, such as Cd vacancies (*V*_Cd_), S vacancies (*V*_S_), Se vacancies (*V*_Se_), and surface-related dangling bonds. These defects can introduce localized electronic states and unpaired spins near the band gap, thereby generating small local magnetic moments. Coey termed this phenomenon d^0^ ferromagnetism. In QDs, the high surface-to-volume ratio leads to a high density of surface defects.^46,47^ In semiconductor nanocrystals, these localized magnetic moments can interact through the bound magnetic polaron (BMP) mechanism. In this process, charge carriers trapped at defect sites mediate exchange coupling between neighboring magnetic moments. The overlap of these BMPs can give rise to weak ferromagnetic-like behavior, even in the absence of magnetic dopants. In addition, the quantum confinement effect in QDs enhances the long-range interaction between charge carriers and localized magnetic moments.^47,48^ This interaction may partially overcome thermal fluctuations, contributing to the weak ferromagnetic behavior observed in CdSSe QDs at room temperature. Upon doping Cu into the CdSSe host lattice at concentrations ranging from 1% to 3%, the saturation magnetization (*M*_s_) increases from 0.079 emu g^−1^ (for CdSSe:Cu 1%) to approximately 0.11 emu g^−1^ (for CdSSe:Cu 3%), and a magnetic hysteresis loop appears, demonstrating the formation of room-temperature ferromagnetism. This phenomenon is explained by the successful substitution of Cu^2+^ ions for Cd^2+^ ions in the crystal lattice, creating localized magnetic moments associated with the unsaturated electronic states of the Cu^2+^ ions, which act as magnetic centers in the system. The formation of macroscopic magnetic order in this diluted magnetic semiconductor (DMS) system primarily stems from the exchange interaction between the localized spins of Cu ions and the charge carriers of the CdSSe host lattice.

However, when the Cu dopant concentration reaches 5% and 10%, *M*_s_ decreases significantly, indicating the appearance of antiferromagnetic interactions. At low Cu concentrations, isolated Cu^2+^ ions can be successfully incorporated into the CdSSe lattice by substituting Cd^2+^ sites. Since Cu^2+^ possesses an unpaired 3d electron, the number of localized magnetic moments increases, leading to the enhancement of *M*_s_. In addition, Cu incorporation can generate defect states such as Cd vacancies, S/Se vacancies, and surface-related dangling bonds, which mediate exchange interactions through BMP mechanism. The overlap of BMPs becomes more effective at moderate Cu concentration, resulting in the maximum Ms observed for the 3% Cu-doped sample.

However, when the Cu concentration further increases to 5% and 10%, the average distance between Cu ions decreases. As a result, neighboring Cu ions tend to interact through antiferromagnetic Cu–Cu exchange coupling, which partially cancels their magnetic moments and reduces the net magnetization. Moreover, excessive Cu doping may promote defect clustering, lattice distortion, and the formation of non-radiative or magnetically inactive Cu-related complexes. These effects weaken the BMP-mediated ferromagnetic exchange network and disturb the long-range alignment of localized magnetic moments. Consequently, although the total Cu content increases, the effective number of magnetically active centers decreases, leading to the reduction of Ms at higher Cu concentrations.^48–50^ This behavior is similar to that reported for Co-doped CdS.^1^ The magnetic moment of CdS QDs increases with increasing Co concentration and reaches a maximum at 4% Co. Beyond this level, *M*_s_ decreases with further increase in Co content. Upon doping Cu into CdSe,^35^ CdS,^12^ and ZnCdS^50^ systems, authors have also reported that the Cu concentration plays a crucial role in tuning the magnetic properties of the material. This result confirms that the Cu concentration plays a decisive role in tuning and optimizing the spintronic properties of the CdSSe QDs.

## 4. Conclusion

In this study, Cu-doped CdSSe QDs with Cu concentrations ranging from 1 to 10% were successfully synthesized *via* a wet chemical method, and the influence of Cu concentration on the optical and magnetic properties of the material was investigated. The results indicate that the CdSSe:Cu QDs all possess a zinc-blende structure. The lattice constant decreases from 5.96 Å (for the undoped CdSSe QDs) to 5.85 Å (for CdSSe:Cu 10%), demonstrating that Cu^2+^ ions have successfully substituted for Cd^2+^ ions in the CdSSe host lattice. EDX spectra and EDX mapping also confirm the presence of Cd, S, Se, and Cu elements in the CdSSe:Cu QDs. Both the absorption and PL spectra exhibit a blue shift as the Cu concentration increases. The absorption peak blue-shifts from 473 nm to 409 nm, while the emission peak shifts from 558 nm to 468 nm. The PL lifetime increases dramatically from 16.26 ns (for undoped CdSSe) to 882.57 ns (for CdSSe:Cu 10%). This behavior is attributed to the trapping of charge carriers in localized Cu-related d-states located deep within the band gap. The resulting reduction in wavefunction overlap between conduction-band electrons and trapped holes slows the radiative recombination process. Magnetization measurements reveal that the CdSSe:Cu QDs exhibit weak ferromagnetism at room temperature. The magnetization reaches its maximum at a 3% doping level and subsequently decreases at 5% and 10% concentrations due to antiferromagnetic interaction. With long PL lifetimes, tunable emission wavelengths, and adjustable weak ferromagnetism, this material opens up broad application prospects in high-efficiency solar cells, LEDs, photodetectors, and biological labeling.

## Author contributions

N. T. T. Hoan and P. M. Tan: writing – original draft, methodology, experiment, investigation. L. A. Tuyen and N. Q. Hung: methodology, investigation. V. T. Nguyen, V. T. K. Lien: data curation, conceptualization. D. T. Hue, N. T. H. Nga: investigation, formal analysis. P. V. Duong: experiment, methodology, investigation. N. X. Ca: writing – review & editing, methodology, investigation.

## Conflicts of interest

The authors declare that they have no known competing financial interests or personal relationships that could have appeared to influence the work reported in this paper.

## Data Availability

Data will be made available on request.
